# Delayed Use of ChatGPT by Japanese Doctors Due to Limited English Language Proficiency: A Survey-Based Study in Japan

**DOI:** 10.7759/cureus.91443

**Published:** 2025-09-01

**Authors:** Tamotsu Kuribayashi, Takashi Chinen, Shinsuke Nagai, Koichiro Shigeta, Yasushi Fujirai, Hidenori Uda

**Affiliations:** 1 General Medicine, Japanese Red Cross Kagoshima Hospital, Kagoshima, JPN; 2 Clinical Oncology, Outpatient Therapy Center, University of the Ryukyus Hospital, Ginowan, JPN; 3 Gastroenterology, Kirishima Medical Center, Kirishima, JPN; 4 General Practice, Japan Association for Development of Community Medicine, Chiyoda, JPN

**Keywords:** academic writing, artificial intelligence, chatgpt, early career, nonnative english speakers, rural doctors

## Abstract

Background: Artificial intelligence (AI) systems, such as Chat Generative Pretrained Transformer (ChatGPT), can provide efficient medical support for doctors. However, the adoption of ChatGPT may depend on users' proficiency in English. In this study, we surveyed the use of ChatGPT by early-career doctors, especially those working in rural areas of Japan.

Methods: We conducted an anonymous online survey targeting early-career doctors (one to nine years of clinical experience) in Japan. The survey was conducted between October 10 and December 31, 2024. Participants were asked to provide their demographic information, experience using ChatGPT, and the benefits and challenges of using it.

Results: Forty-six physicians completed the questionnaire. Of the respondents, 32.6% (15/46) had never used ChatGPT. Among the users, only 12.9% (4/31) began using the English version of ChatGPT before the Japanese version became available. Supporting the writing of academic papers in English was the most frequently cited benefit of ChatGPT among respondents, as reported by 18 users.

Discussion: Our findings suggest a potential link between limited English proficiency and delayed initial adoption of ChatGPT among early-career Japanese doctors. Nevertheless, they supported Japanese doctors in their academic writing. These findings underscore the need to introduce targeted efforts to promote ChatGPT, particularly in non-English-speaking communities. Although AI is associated with some ethical concerns, this study highlights the utility of ChatGPT for this population.

Conclusion: Our findings suggest a potential link between limited English proficiency and the delayed initial adoption of ChatGPT among early-career Japanese doctors.

## Introduction

Artificial intelligence (AI) can provide doctors with efficient medical advice [1]. One well-known generative AI tool is Chat Generative Pretrained Transformer (ChatGPT), which was launched by OpenAI (San Francisco, CA) on November 30, 2022. Its use has rapidly expanded to the field of medicine. ChatGPT is applicable in research, clinical practice, and medical education [2,3]. For example, ChatGPT enhances communication skills by simulating patient interactions [4]. Moreover, this tool offers a customized learning experience based on learner requirements and provides medical information concerning clinical decision-making [5]. Clinicians can use these aspects of ChatGPT.

The use of AI as a mentor in medical education has recently attracted attention [6,7]. A previous randomized clinical trial showed that the AI tutor system demonstrated superior performance compared with remote expert instructions [8]. AI can improve medical systems in rural areas where doctors work and have few opportunities for clinical education and mentoring [9]. Only practitioners have few mentors and limited training opportunities, particularly in rural areas where medical resources are scarce. However, nonnative English speakers sometimes have difficulty accessing information written in English [10]. A previous study in a non-native English-speaking country reported that approximately 30% of doctors used ChatGPT in July 2023 [11]. In Japan, support for the Japanese version of ChatGPT was delayed by six months after its first launch. Further, the global trend in publications related to ChatGPT in Japan lags behind that of other countries [12]. ChatGPT has the potential to act as a mentor for rural doctors who often lack immediate access to specialists and peer consultations. However, the use of ChatGPT by early-career doctors in rural areas remains unclear.

The primary aim of this study was, therefore, to survey the usage of ChatGPT before and after it became available to early-career doctors in Japan, particularly those with opportunities to work in rural areas. Second, we examined the possibility of using ChatGPT as a training tool to enhance academic writing skills.

## Materials and methods

Study design

This was a questionnaire-based, cross-sectional survey.

Ethical considerations

This study was approved by the Institutional Review Board of the Japanese Red Cross Kagoshima Hospital (July 2, 2024).

Setting, participants, and data sources

The study sample consisted of doctors with one to nine years of clinical experience in Japan. There were no exclusion criteria. We sent e-mails to doctors who had graduated from Jichi Medical University (Shimotsuke, Japan) within the past nine years via the branch chief of each prefecture belonging to the Japan Association for Development of Community Medicine, an organization established to secure community medicine. Jichi Medical University graduates are required to work in remote areas within nine years of graduation and are scattered throughout all prefectures. This graduate population has a fundamentally high need for mentors and is representative of the overall situation in Japan. The e-mails included a brief background on the study and information on the length of time required to answer the questions, objectives of the study, and details of the survey conducted between October 10 and December 31, 2024. We sent reminder e-mails to the participants at one-month intervals after the initial invitation. The respondents were asked to participate in an open, voluntary, and anonymous survey. They were allowed to review and change their answers while responding and were allowed to answer once. The participants did not receive any incentives. All responses were automatically saved using Google Drive (Google LLC, Mountain View, CA).

Questionnaire

We conducted an anonymous online survey using Google Forms (Google LLC). The questionnaire was tested in Japanese and did not undergo any formal validation or reliability testing; however, its usability and technical functionality were confirmed by the study members in advance. The survey consisted of three sections with 11 questions (Table 1) extracted from a previous study [13] that conducted a systematic search on the utility and limitations of ChatGPT in healthcare education, research, and practice. The first section consisted of six questions about the participants’ demographics, including clinical experience, major specialties, status, and experience using ChatGPT. The second section consisted of one question asking about the disadvantages of ChatGPT cited in the study [13] for participants who had never used it (i.e., non-ChatGPT users). The third section for participants who had used ChatGPT (i.e., ChatGPT users) consisted of four questions about the benefits achieved from each of the clinical, research, and education fields to comprehensively cover their use and disadvantages, as in the second section.

**Table 1:** Questionnaire

Questionnaire
Section 1
1) Is this your first time answering this survey?
If you choose “Yes”, you will proceed to Question 2.
If you choose “No”, you will end this survey.
2) How many years have passed since your graduation?
3) Please select your specialty:
1. Junior Resident, 2. Internal Medicine, 3. General Medicine, 4. Emergency Medicine, 5. Pediatrics, 6. Dermatology, 7. Psychiatry, 8. Surgery, 9. Orthopedics, 10. Obstetrics and Gynecology, 11. Ophthalmology, 12. Otolaryngology, 13. Urology, 14. Neurosurgery, 15. Anesthesiology, 16. Radiology, 17. Pathology, 18. Clinical Laboratory Medicine, 19. Plastic Surgery, 20. Rehabilitation Medicine, 21. Others
4) What is your current employment status?
1. University hospital, 2. Non-university hospital, 3. Clinic with beds, 4. Clinic without beds, 5. Others
5) What is your current role at your institution?
1. Junior Resident, 2. Trainee, 3. Staff, 4. Graduate student, 5. Director
6) Have you ever used ChatGPT?
If you choose “Yes”, you will proceed to Questions 8, 9, 10, 11
If you choose “No”, you will proceed to Question 7
Section 2
7) What challenges do you see with using ChatGPT? (Select up to 3 options)
1. Ethical concerns (e.g., bias, plagiarism, copyright infringement)
2. Potential for incorrect answers
3. Lack of transparency regarding sources and reasoning in answers
4. Hindering personal growth
5. Concerns regarding data security
Section 3
8) When did you start using ChatGPT?
1. November 2022 (ChatGPT released) - April 2023
2. May 2023 (ChatGPT supported for Japanese) - April 2024
3. May 2024 or later
9) How often do you use ChatGPT?
1. Daily
2. Several times a week
3. Several times a month
4. I have used it, but not regularly
10) What benefits do you see in using ChatGPT? (Select up to 3 options based on actual use)
1. Creating similar clinical cases
2. Providing feedback comments for students
3. Enhancing communication skills
4. Supporting in writing academic papers in English
5. Generating queries for comprehensive systematic reviews
6. Enhancing research processes (e.g., generating computer codes or conducting comprehensive literature reviews)
7. Refining personalized medicine, diagnostic ability, and treatment quality
8. Improving patients’ health literacy
9. Producing referral letters or discharge summaries in clinical practice
10. No applicable benefit 1
11. No applicable benefit 2
11) What challenges do you see in using ChatGPT? (Select up to 3 options)
1. Ethical concerns (e.g., bias, plagiarism, copyright infringement)
2. Potential for incorrect answers
3. Lack of transparency regarding sources and reasoning in answers
4. Hindering personal growth
5. Concerns regarding data security

Data analysis

Data were collected using Google Forms, and all analyses were performed using Microsoft Excel (Microsoft Corporation, Redmond, WA). No data were missing. The sample size was not predetermined due to the lack of sufficient prior research targeting doctors in rural areas.

## Results

Of the 162 physicians invited to participate, 46 (28.3%) completed the survey. Of these, 31 (67.3%) used ChatGPT. The most frequently selected respondents were internal medicine doctors, trainees, and nonuniversity hospital doctors (Table 2).

**Table 2:** Participant information IQR: interquartile range

Participants	ChatGPT users	ChatGPT nonusers
N	31	15
Years after graduation, median (IQR)	6 (5-8)	6 (2.5-7)
Characteristic of each group, n (%)		
Speciality		
Junior Resident	3 (9.7)	4 (26.7)
Internal Medicine	13 (41.9)	3 (20)
General Medicine	7 (22.6)	2 (13.3)
Emergency Medicine	3 (9.7)	1 (6.7)
Pediatrics	1 (3.2)	0 (0)
Psychiatry	1 (3.2)	1 (6.7)
Surgery	1 (3.2)	0 (0)
Orthopedics	0 (0)	1 (6.7)
Obstetrics and Gynecology	1 (3.2)	2 (13.3)
Others	1 (3.2)	1 (6.7)
Employment status		
Junior Resident	3 (9.7)	4 (26.7)
Trainee	13 (41.9)	8 (53.3)
Staff	10 (32.3)	3 (20)
Graduate student	1 (3.2)	0 (0)
Director	4 (12.9)	0 (0)
Institution		
University hospital	6 (19.4)	1 (6.7)
Non-university hospital	18 (58.1)	11 (73.3)
Clinic with beds	1 (3.2)	0 (0)
Clinic without beds	4 (12.9)	1 (6.7)
Others	2 (6.5)	2 (13.3)

**Figure 1:**
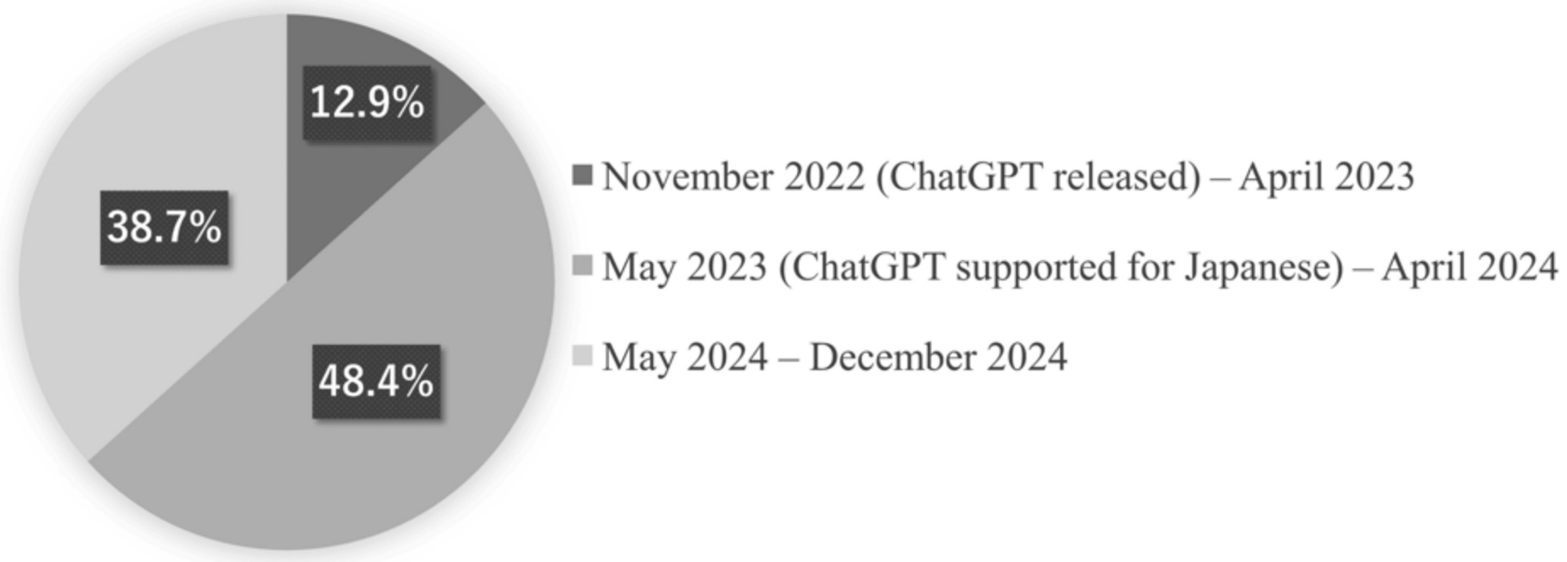
Starting date of ChatGPT use We asked ChatGPT users when they first began using the tool. The ChatGPT usage period was divided into three parts. The first phase began in November 2022 when ChatGPT was first released: 12.9% (4/31). The second phase began in May 2023, when the Japanese version was released: 48.4% (15/31). The final phase began in May 2024, one year after the release of the Japanese version: 38.7% (of 12/31)

**Figure 2:**
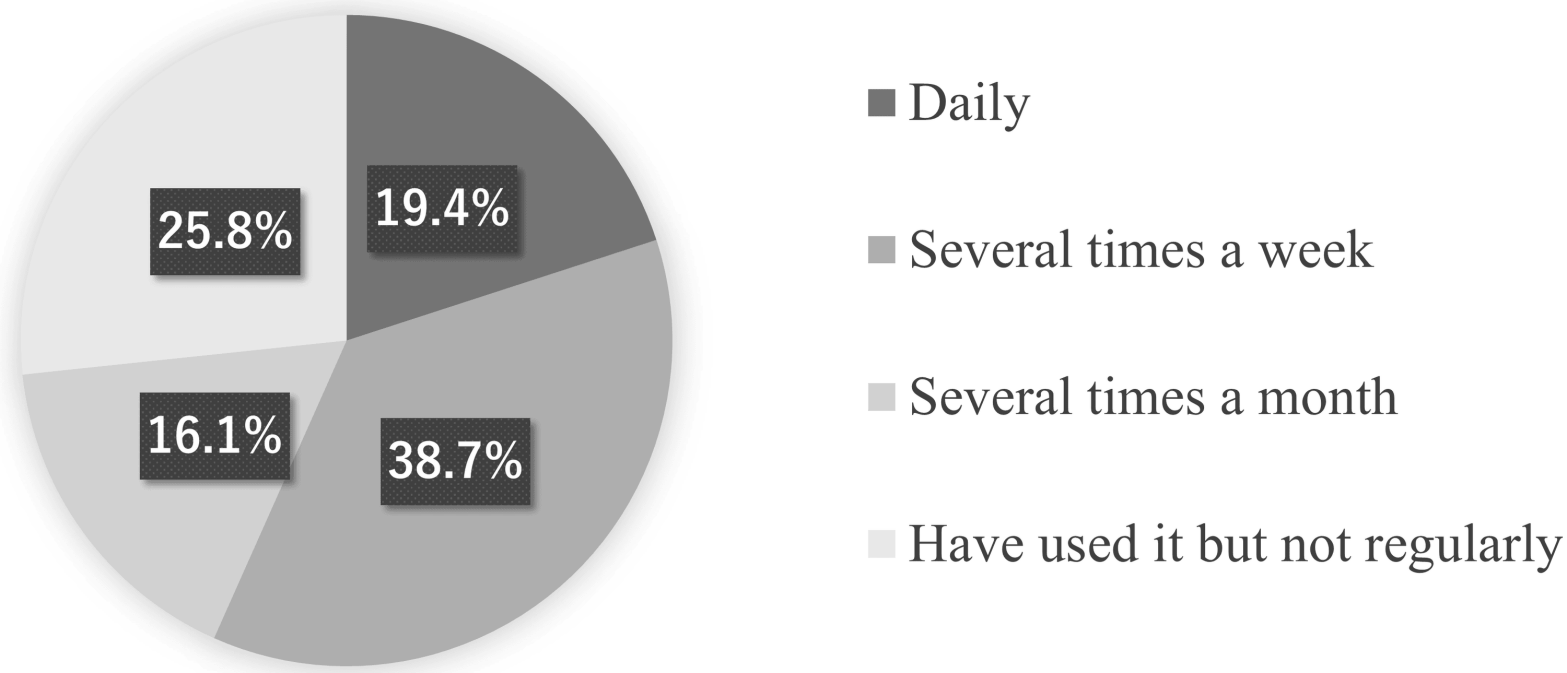
Overall frequency of ChatGPT use The frequency of ChatGPT use was divided into four categories: daily, 19.4% (6/31); several times per week, 38.7% (12/31); several times per month, 16.1% (5/31); and not regularly, 25.8% (8/31)

Among ChatGPT users, 12.9% (4/31) of the respondents started using ChatGPT before the Japanese version was released (Figure 1). Furthermore, 58.1% (18/31) of ChatGPT users accessed it several times a week or daily (Figure 2).

The benefits of using ChatGPT among the respondents who used this tool are shown in Table 3. Supporting the writing of academic papers in English was the most common benefit for respondents, as cited by 18 users. Providing comments to students was the least selected option.

**Table 3:** Benefits of using ChatGPT We investigated the three most prioritized benefits for ChatGPT users. If respondents identified fewer than three advantages, they had the option to select “No applicable benefit” (questions 10 and 11). We divided the count for each item by three times the number of ChatGPT users

Benefits	n (%)
1.Creating similar clinical cases	7 (7.5)
2. Providing feedback comments for students	1 (1.1)
3. Enhancing communication skills	8 (8.6)
4. Supporting in writing academic papers in English	18 (19.4)
5. Generating queries for comprehensive systematic reviews	2 (2.2)
6. Enhancing research processes (e.g., generating computer codes or conducting comprehensive literature reviews)	10 (10.8)
7. Refining personalized medicine, diagnostic ability, and treatment quality	13 (14.0)
8. Improving patients health literacy	1 (1.1)
9. Producing referral letters or discharge summaries in clinical practice	3 (3.2)
10. No applicable benefit 1	18 (19.4)
11. No applicable benefit 2	12 (12.9)

Table 4 presents the challenges of using ChatGPT for all the respondents. There were no significant differences between ChatGPT users and nonusers. The potential for incorrect answers was the most frequently selected item among ChatGPT users. Lack of transparency in answers and the potential for incorrect answers were the most frequently selected items among nonusers.

**Table 4:** Challenges in using ChatGPT for all respondents We investigated the three most prioritized challenges and divided the count of each item by three times the number of ChatGPT users and nonusers

Challenges	ChatGPT users, n (%)	ChatGPT nonusers, n (%)
1. Ethical concerns (e.g., bias, plagiarism, copyright infringement)	18 (19.4)	10 (22.2)
2. Potential for incorrect answers	30 (32.3)	14 (31.1)
3. Lack of transparency regarding sources and reasoning in answers	23 (24.7)	14 (31.1)
4. Hindering personal growth	10 (10.8)	3 (6.7)
5. Concerns regarding data security	12(12.9)	4 (8.9)

## Discussion

Principal findings

In this study, we surveyed the usage of ChatGPT before and after it became available to early-career doctors in Japan. Based on a survey, we found that only 13% of ChatGPT users among early-career Japanese doctors began using it before the Japanese version was launched. This percentage is lower than that of healthcare workers in other countries [14,15]. This delayed adoption could be associated with Japan’s low English proficiency levels [16], as many Japanese doctors hesitate to use English because of language concerns. Despite the broad spread of ChatGPT, over 30% of the respondents had never used it. The use of ChatGPT among early-career doctors increased with the launch of the Japanese version.

Furthermore, ChatGPT is primarily used to support academic English writing. Some users perceived the tool as a mentor rather than just a writing assistant. Some studies have reported the benefits of ChatGPT in writing English publications for nonnative English speakers [17,18]. Early-career doctors, particularly those with opportunities to work in rural areas, may have fewer opportunities to learn academic writing in English. ChatGPT can revise publications written by nonnative English speakers, which is beneficial for early-career doctors in Japan. A prior study in China reported that ChatGPT demonstrated superior performance compared to interprofessional mentors in generating educational clinical scenarios [19]. The utility of ChatGPT, other than supporting academic writing in nonnative English-speaking countries, has recently attracted attention. However, the integration of AI into mentorship programs has raised several concerns. Responses from ChatGPT may be false and produce biases. Further, as an ethical issue, using patient data for training ChatGPT can invade privacy [13]. The utility of ChatGPT for early-career doctors in rural areas outweighs these concerns. In the case of concerns about ChatGPT, such as ethical issues and the unreliability of responses, relying on real specialists is the best alternative. Moreover, this survey can provide insights into the spread of ChatGPT among nonnative English speakers in rural areas. While several efforts to promote medical AI by the public and private are anticipated in Japan [20], the introduction of AI into medical fields in Japan has lagged behind other developed countries [21], as this study showed that the adoption of ChatGPT is delayed among early-career doctors in Japan.

Limitations of the study

This study had several limitations. This study surveyed the current usage patterns and perceived utility of ChatGPT among early-career doctors in Japan; thus, the sample size of this study was limited to explore the overall situation in Japan. Therefore, the generalizability of our findings to a broader population of early-career doctors in Japan may be limited. Further, our sample may have been biased toward doctors who already had an interest in ChatGPT, as the survey was stated as targeting “ChatGPT in self-training among early career doctors.” To address this potential selection bias, this survey specifically targeted a representative sample from across Japan, not just individuals with a preexisting interest in ChatGPT, to eliminate selection bias. Furthermore, response bias may have occurred because of the structure of the questionnaire, particularly if participants tended to choose extreme options regarding the benefits of ChatGPT (11 items). In addition, we could not precisely assess the participants' English proficiency; thus, the relationship between ChatGPT adoption and users’ English skills should be evaluated with caution. Finally, the numbers assigned to the questionnaire options were nominal and not ordinal; therefore, the exact validity of the questions could not be assessed. Moreover, we did not perform formal validation or reliability testing of our questionnaire due to the scarcity of prior research on this specific population. Nevertheless, we ensured validity by extracting three items from each of the clinical, research, and educational fields based on a previous systematic review [13], thereby providing comprehensive coverage.

## Conclusions

This study found that the adoption of ChatGPT among early-career Japanese doctors lagged behind that of doctors in other countries, likely because of limited English proficiency. However, the release of the Japanese version of this resource led to an increase in its use. Currently, this tool is primarily used for English academic writing. ChatGPT effectively supports rural doctors with limited opportunities to undergo academic training. Although there are concerns regarding AI-generated false information and ethical issues, the utility of ChatGPT for this population appears to overcome these risks. Our study highlights the delayed adoption of ChatGPT in Japan and underscores the need for targeted efforts to promote it, particularly in non-English-speaking communities.
